# Work-related experiences in intensive and palliative care units and their relation to burnout

**DOI:** 10.1186/2197-425X-3-S1-A649

**Published:** 2015-10-01

**Authors:** C Teixeira, SM Pereira, P Hernández-Marrero, AS Carvalho

**Affiliations:** Instituto de Ciências Biomédicas Dr. Abel Salazar, Universidade do Porto, Porto, Portugal; Departamento de Anestesia e Cuidados Intensivos, Centro Hospitalar do Porto, Hospital de Santo António, Porto, Portugal; Gabinete de Investigação em Bioética, Instituto de Bioética, Universidade Católica Portuguesa, Porto, Portugal; Departamento de Enfermería, Universidad de Las Palmas de Gran Canaria, Facultad de Ciencias de la Salud, Las Palmas, Spain

## Introduction

Professionals working in intensive and palliative care units and caring for patients at the end of life are at risk of developing burnout. Literature shows that work-related experiences are determinant factors to develop the burnout syndrome.

## Objectives

To identify which work-related experiences are significantly associated with burnout among healthcare professionals who provide end-of-life care in intensive and palliative care units in Portugal.

## Methods

Multicenter quantitative, comparative study. A survey study was conducted using: The Maslach Burnout Inventory and a questionnaire including a set of work-related experiences. 355 professionals from intensive and palliative care units were included in this study. Univariate and multivariate logistic regression analyses were performed; OR sidelong with 95% of CI were calculated.

## Results

Out of the 355 professionals included in this study, 27% were in burnout (this defined as being in burnout and in high risk of developing this syndrome). Univariate regression analyses showed that higher burnout levels were significantly associated with the following work-related experiences: night shifts, conflicts, decisions to withhold treatment, decisions to withdraw treatment and implementing terminal sedation. When controlling for socio-demographic and educational characteristics of the participant professionals, and for the setting (intensive vs. palliative care units), the only variable that remained significantly associated to higher burnout levels was ´conflicts´.

## Conclusions

Work-related experiences increase the risk of developing burnout among professionals who provide end-of-life care in intensive and palliative care units. Experiencing conflicts in the workplace was the most significant variable associated to higher burnout levels. These findings suggest that team-dynamics and conflict-management are paramount in the implementation of strategies and programs aiming at preventing or minimizing burnout.

## References

[1] Embriaco N, Azoulay E, Barrau K, Kentish N, Pochard F, Loundou A, Papazian L (2007). “High level of burnout in intensivists”. Am J Respir Crit Care Med.

[2] Maslach C, Schaufeli WB, Leiter MP (2001). “Job burnout”. Annu Rev Psychol.

[3] Ponet M, Toullic P, Papazian L, Kentish-Barnes N, Timsit JF, Pochard F, Chevret S, Schlemmer B, Azoulay E (2007). “Burnout syndrome in critical care nursing staff”. Am J Respir Crit Care Med.

[4] Teixeira C, Ribeiro O, Fonseca AM, Carvalho AS: Ethical decision making in intensive care units: a burnout risk factor? Results from a multicenter study conducted with physicians and nurses. Journal of Medical Ethics. 10.1136/medethics-2012-100619 23408707

